# Topical 5-Fluorouracil 0.5% as primary treatment for Ocular Surface Squamous Neoplasia

**DOI:** 10.1177/11206721241256687

**Published:** 2024-05-23

**Authors:** Gabriela Carneiro Teixeira, Mateus Inacio Lemes de Resende, Melina Correia Morales, Arthur Gustavo Fernandes

**Affiliations:** 1 Department of Visual Sciences and Ophthalmology, Federal University of São Paulo, São Paulo, SP, Brazil; 2 Department of Anthropology and Archaeology, 2129 University of Calgary, Calgary, AB, Canada

**Keywords:** conjunctival tumor, topical chemotherapy, ocular oncology, ocular surface, ophthalmology

## Abstract

**Purpose:**

To evaluate the efficacy of topical treatment with 5-Fluorouracil (5-FU) 0.5% in cases of Ocular Surface Squamous Neoplasia (OSSN), and to assess the tolerance of patients undergoing treatment.

**Methods:**

Patients with clinical diagnosis of OSSN referred to the Ocular Oncology division from the Federal University of Sao Paulo, Brazil, were recruited for the current study. Patients were treated with topical 5-FU 0.5% using a regimen of 4 times daily for 10 days, followed by a 3-week drug holiday, continued up to 3 cycles before an alternative treatment. Lesions were evaluated at baseline and throughout treatment. Treatment adherence was assessed using the Morisky Medication Adherence scale. Any adverse events along the treatment were noted.

**Results:**

A total of 30 eyes of 30 patients adherent to the treatment were included in the study. Among the total cases treated with 5-FU 0.5%, 24 patients achieved therapeutic success after a mean treatment duration of 21.71 ± 7.77 days, representing a success rate of 80.00% (95% CI: 60.75–91.18%). For each 1 mm2 increase in the lesion area, the odds of treatment success decrease by 6% (OR: 0.94; 95%CI: 0.88–0.99; p = 0.033). Only mild adverse events such as ocular discomfort, ocular burning and tearing were observed along the treatment in 8 patients.

**Conclusions:**

Topical 5-FU 0.5% is an effective therapeutic option in the treatment of OSSN, with an 80% therapeutic success rate, showing good tolerability. The size of the lesion was identified as a factor influencing treatment success, therefore it should be taken into consideration when defining treatment approaches.

## Introduction

Ocular surface squamous neoplasias (OSSN) are the most common non-pigmented malignant neoplasms of the ocular surface, with estimated incidences ranging from 0.13 to 1.9 cases per 100,000 persons, with a higher incidence in equatorial regions and older white males.^1,2^ Risk factors include sun exposure, smoking, and systemic conditions associated with immunosuppression, such as human immunodeficiency virus (HIV) infection and xeroderma pigmentosum.^3^

The gold standard treatment for OSSN is the excision of the lesion with safety margins, associated with cryotherapy on the remaining conjunctival margins. Surgery has the advantage of being not only therapeutic but also diagnostic, enabling the histopathological evaluation of the lesion and its degree of cellular differentiation.^4^ However, surgery may be associated with complications such as limbal insufficiency, leukomas, as well as incomplete excision with compromised margins and recurrence, with rates ranging from 5 to 69% in different studies.^4,5^

The development of topical chemotherapeutics, such as mitomycin C (MMC), interferon alpha 2b (IFN), and 5-fluorouracil (5FU), has increased the therapeutic options for these lesions. They can be used as adjuvant after surgery and as a first-line treatment with good results, as they can treat the entire ocular surface and reduce damage to the limbus, which is associated with more aggressive therapeutic options.^6^

5-fluorouracil is a pyrimidine analog chemotherapeutic that blocks thymidine synthase, thus inhibiting the formation of DNA and RNA, causing cell death.^5^ When used at the concentration of 1%, it has adverse effects such as conjunctival hyperemia, ocular discomfort upon instillation, eyelid edema, and more severe effects, such as superficial keratitis, filamentary keratitis, and stromal melting since 5FU also inhibits proliferation of epithelial cells and fibroblasts.^7^ Despite its side effects, it is better tolerated than mitomycin C, demonstrates similar efficacy to topical IFN, and has the advantage of a lower cost.^4^ The efficacy of 5-FU 1% as the primary therapy for OSSN has been proven with the recommended administration four times daily for one week, followed by three weeks of pause, and the cycle could be repeated up to four times. Following this approach, 82% of patients achieve complete resolution of the lesion with topical treatment and 61% experience at least one medication related side effect.^8^

While the 5-FU at a concentration of 1% was proven as an efficient topical treatment option for OSSN, its side effects may still reduce patient adherence. The purpose of the current study was to evaluate the efficacy and tolerance of topical treatment for OSSN with 5-FU at a reduced concentration of 0.5%, identifying possible adverse effects of the medication.

## Methods

Patients with clinical diagnosis of ocular surface squamous neoplasia referred to the Ocular Oncology division from the Department of Ophthalmology and Visual Sciences of the Federal University of Sao Paulo, Brazil, were prospectively selected to participate in the current study. Those aged less than 18 years old, with previous ocular treatment, with diagnosis of Xeroderma Pigmentosum or immunosuppression conditions such as HIV, and tumours with scleral or corneal infiltration confirmed by Ultrasound Biomicroscopy (UBM) were excluded. The study protocol was approved by the Federal University of Sao Paulo Review Boards and was carried out in accordance with the tenets of the Declaration of Helsinki. Informed consent was obtained from all participants.

All patients were treated with topical 5-FU at a concentration of 0.5%. A comprehensive ocular evaluation including anterior segment photograph was performed at baseline before the treatment.

Lesions were photographed using a DC-3 digital camera (Topcon, Tokyo, Japan), attached to the slit lamp, with 10× magnification focusing on the lesion, after instillation of 1 drop of anesthetic eye drop (proxymetacaine hydrochloride 5 mg/ml; Alcon Laboratórios do Brasil, SP, Brazil) and one drop of toluidine blue (TB) 10 mg/ml (Eye Pharma, SP, Brazil). TB was applied for staining of the ocular surface lesion and margins allowing a precise measurement of the lesion area.^9,10^ Slit lamp photos were taken 1 min after installation of TB eye drop. Images obtained at the slit lamp were measured using the ImageJ application (developed by Wayne Rasband at the National Institute of Mental Health, USA, in Java language), adjusting the scale according to the manufacturer's instructions.^10^

The 5-FU 0.5% drops were prescribed 4 times daily for 10 days, followed by a drug holiday for 3 weeks. After the first cycle of the drug therapy, the patients were re-evaluated to investigate treatment adherence and tolerability.

Treatment adherence was evaluated using the Morisky Medication Adherence (MMAS-8), a scale which evaluates medication-taking behavior and assess patient adherence.^11^ The scale provides better understanding of both unintentional and intentional (such as adverse effects) factors that may contribute to the patient's adherence to the treatment prescribed. Patients with score ≤7 were considered non-adherents and therefore excluded from the study and referred to an alternative treatment.

The monthly cycle of 10 days treatment followed by a 3 week break was continued up to 3 times. Cases with no resolution after the 3 cycles or those with severe adverse events at any treatment point had the drops regimen discontinued and were referred to an alternative treatment.

Data were analyzed using the STATA 14.0 software (StataCorp LP, College Station, TX, USA). Frequency tables were used for descriptive analyses. Factors associated with treatment success were investigated by Firth multiple logistic regression. For all tests, a significant p value was considered when less than or equal to 0.05.

## Results

A total of 30 eyes of 30 patients adherent to the prescribed treatment were included in the study. Table 1 shows the patients baseline characteristics.

**Table 1.** Patient's baseline characteristics.

Variable	
Sex, N(%)	
Males	22 (73.33)
Females	8 (26.67)
Age, mean ± sd (median)	69.08 ± 9.00 (68.04)
Location, N(%)	
Perilimbar	16 (53.33)
Conjunctiva	14 (46.67)
Lesion area in mm^2^, mean ± sd (median)	5.82 ± 3.22 (5.63)

**Figure 1.**
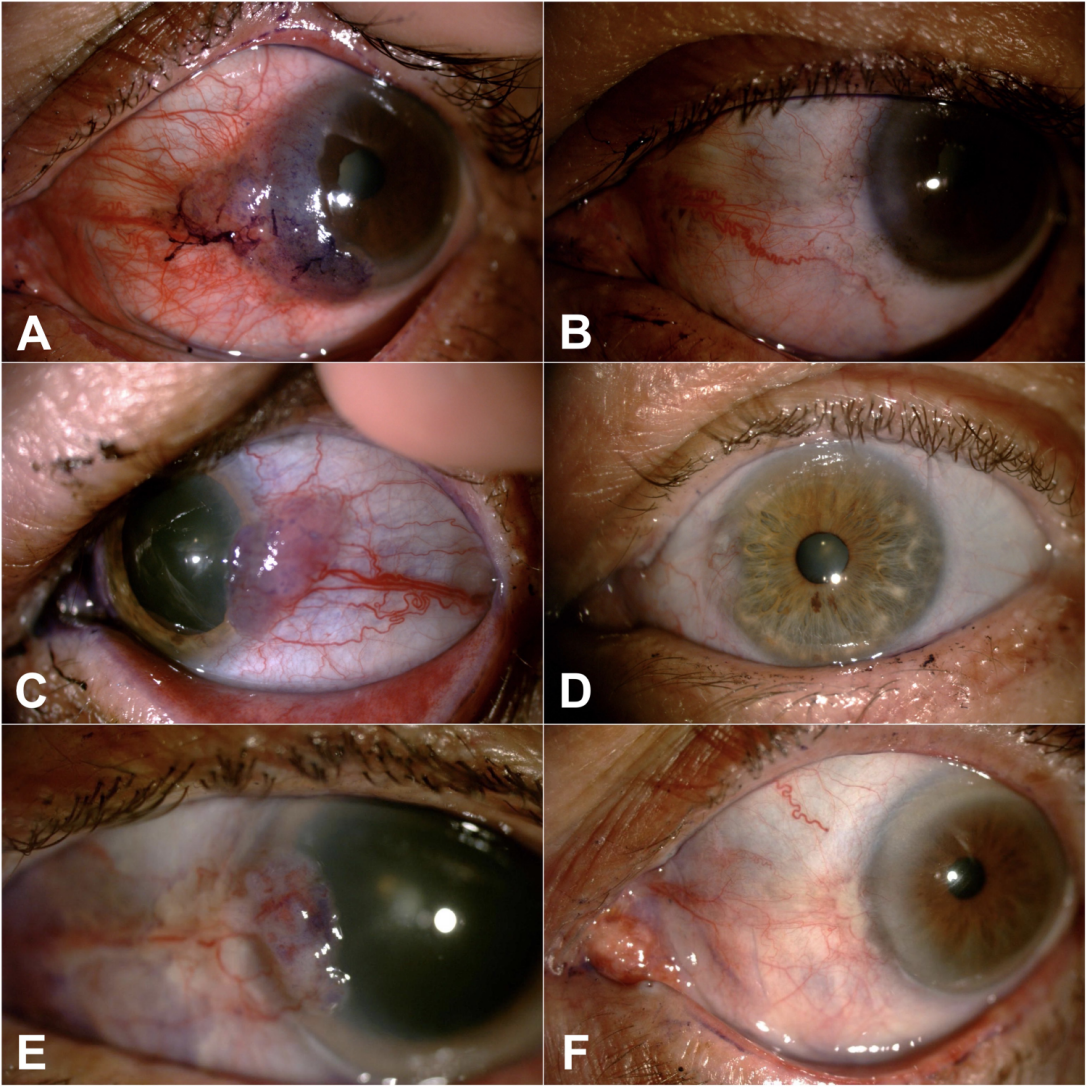
72-year old male patient clinically diagnosed with OSSN in the left eye (A) presenting complete resolution of the lesion after 2 cycles of treatment with 5-FU 0.5% eyedrops (B). 73-year old male patient clinically diagnosed with OSSN in the left eye (C) presenting complete resolution of the lesion after 2 cycles of treatment with 5-FU 0.5% eyedrops (D). 79-year old male patient clinically diagnosed with OSSN in the left eye (E) presenting complete resolution of the lesion after 2 cycles of treatment with 5-FU 0.5% eyedrops (F).

Among all cases treated with 5-FU 0.5%, 24 patients achieved therapeutic success after a mean treatment duration of 21.71 ± 7.77 days of medication use, representing a success rate of 80.00% (95% CI: 60.75–91.18%). Figure 1 illustrates 3 successful cases after treatment with 0.5% 5-FU. Among the cases of failure, 4 were referred for surgical excision, 1 case was referred for treatment with IFN, and 1 case was referred for treatment with MMC.

Table 2 shows the Firth logistic regression analysis applied to investigate factors associated with successful cases.

**Table 2.** Firth logistic regression analysis for successful cases.

Variable	Odds Ratio (95%Confidence Interval)	p-value
Age	0.92 (0.79–1.08)	0.314
Location		
Perilimbar	Reference	—
Conjunctiva	0.52 (0.05–5.26)	0.582
Lesion area in mm^2^	0.94 (0.88–0.99)	0.033

There was no significant effect of age or location on the outcomes (p < 0.05). There was a statistically significant effect of lesion area on the treatment outcome. For each 1 mm2 increase in the lesion area, the odds of treatment success with 5-FU 0.5% decrease by 6% (OR: 0.94; 95%CI: 0.88–0.99; p = 0.033).

Along the treatment course, 8 patients (26.7%) reported mild adverse effects. The self-reported events included ocular discomfort (3), ocular burning (3), tearing (1), and palpebral burning (1). None of the cases needed to suspend the treatment due to these mild events. Despite the adverse events, all the 8 cases showed a successful resolution after treatment.

## Discussion

The first report of use of 5-FU 1% eye drops for OSSN treatment was published by de Keizer in 1986, in patients with premalignant lesions of the cornea and conjunctiva.^12^ 5-FU was already considered an efficient treatment for premalignant and malignant epithelial lesions of the skin, and it was also used in the eye to slow down the growth of fibroblasts and scar formation in filtering blebs.^12^ Since then, various regimens of treatment with topical 5-FU 1% as a primary treatment have been studied, with different frequencies of administration, duration and number of cycles prescribed, resulting in different success rates. A study performed by Joag et al. in 2016 with 44 patients diagnosed with OSSN who were treated primarily with 5-FU 1% 4 times daily for one week followed by a drug holiday for 3 weeks for as many cycles until clinical resolution showed complete resolution of the lesion in 82% of cases.^8^ Similar rates were observed by Parrozzani et al in 2017 who have reported a success rate of 83% with similar treatment regimen.^5^ A recent study published in 2023 by Wylegala in 2023 with 258 eyes with OSSN submitted to primary treatment with 5-FU 1% with a regimen of 4 times daily for one week follow by a three-week drug holiday, continued until tumor resolution, has shown a success rate of 87% of the patients after a mean number of 4 cycles of medication.^13^ Our study, the first to evaluate the efficacy of 5-FU at a reduced concentration of 0.5% as primary treatment for OSSN, showed a success rate of 80%, which is similar to 5-FU 1% success rates in previous studies.

Regarding side effects, most studies have reported mostly mild side effects following treatment with 5-FU 1%, however, the Wylegala's study in 2023 has observed 3% of individuals developing severe keratopathy and 3% developing ocular surface infections while using 5-FU 1% (Pseudomonas aeruginosa keratitis, bacterial conjunctivitis, and Herpes simplex keratitis).^13^ On the same study, another 3% of the patients developed limbal stem cell deficiency. Joag et al has reported that 61% of the patients presented at least one side effect following 5-FU 1% treatment, most of them pain (39%), followed by tearing (23%), and one patient developed infection.^8^ Parrozzani in 2017 has documented low-to-mild side effects in 48% of the 5-FU 1% treated patients, however there were no severe side effects and no patient discontinued therapy due to side effects.^5^ Our study using reduced concentration of 5-FU resulted in a better side effect profile when compared to these studies on 5-FU 1%, maintaining treatment efficacy. Only 26,7% of patients reported side effects, all of them characterized as mild, therefore, no patient needed to discontinue treatment due to discomfort.

Other topical agents available for OSSN treatment are IFN and MMC. Topical IFN eye drops present minimal side effects, however when administered as subconjunctival injections it can cause a flu-like syndrome.^4^ IFN is known to be more tolerable than 5-FU 1% due to its minimal side effect profile, such as hyperemia, and, based on the findings of our study, we believe 5-FU 0.5% presents a similar tolerability, since only mild symptoms were observed in our cases. MMC, on the other hand, has more severe side effects reported, such as pain, epitheliopathy, ectropion and punctal stenosis, and it is considered less tolerable than both IFN and 5-FU.

The population of our study consists of patients of the Brazilian public health system, the Sistema Único de Saúde (SUS), with a high frequency of individuals with low income and low education levels, both factors that may contribute to low adherence to proposed treatments. Therefore, the MMAS-8 scale was applied and only the patients which were considered adherent to the treatment based on its responses were included in our study, avoiding potential bias associated with the irregular use of medication. In some cases, surgical treatment was indicated as the first option when the patient had important impediments to topical treatment, such as difficulty to attend to the ambulatory appointments, or to purchase the medication. 5-FU 0.5% has a lower cost when compared to IFN and MMC, it does not need to be kept refrigerated, and it causes mild or no side effects, characteristics that support it as a first-line treatment option for OSSN and facilitate adherence.

Our study presents promising results regarding the use of 5-FU at a reduced concentration of 0.5% as an efficient and safe treatment option for OSSN, however, it is important to note some limitations such as sample size, lack of control group, and no longitudinal arm. We recommend continued studies with a larger number of patients, the use of anterior segment OCT to precisely evaluate changes on the conjunctival epithelia, long-term follow-up in order to evaluate recurrence rates, and studies comparing 5-FU 0.5% to other treatment options, such as IFN, MMC, and 5-FU 1%, to reinforce our results.

In conclusion, the 5-FU 0.5% is an effective therapeutic option in the treatment of OSSNs, with an 80% therapeutic success rate in the study, showing good tolerability. The lesion area was identified as a factor influencing treatment success, therefore it should be taken into consideration when defining the treatment of choice. Further studies with a larger number of patients and a longer follow-up are important to validate our results and identify recurrence rates after treatment.
